# Pivoting to Video Telehealth for Delivery of HIV Care During COVID-19: A Brief Report

**DOI:** 10.1089/tmr.2021.0010

**Published:** 2021-08-06

**Authors:** Gabrielle F. Gloston, Giselle A. Day, Hilary N. Touchett, Kathy E. Marchant-Miros, Julianna B. Hogan, Patricia V. Chen, Amber B. Amspoker, Terri L. Fletcher, Thomas P. Giordano, Jan A. Lindsay

**Affiliations:** ^1^ Houston VA HSR&D Center for Innovations in Quality, Effectiveness and Safety, Michael E. DeBakey VA Medical Center, Houston, Texas, USA.; ^2^ Menninger Department of Psychiatry and Behavioral Sciences, Baylor College of Medicine, Houston, Texas, USA.; ^3^ VA South Central Mental Illness Research, Education and Clinical Center, A Virtual Center, Houston, Texas, USA.; ^4^ Department of Medicine, Baylor College of Medicine, Houston, Texas, USA.

**Keywords:** COVID-19, HIV, telemedicine, veterans

## Abstract

**Background:** The rapid spread of the SARS-CoV-2 pandemic obstructed human subjects research, including our own randomized hybrid type 2 effectiveness–implementation trial comparing multidisciplinary HIV care delivered by video telehealth to home (VTH) versus in-person delivery.

**Methods:** Given the Veteran Health Administration's extensive telehealth infrastructure and our team's expertise in personalized implementation of virtual treatments (PIVOT), we shifted our focus to meet the immediate needs of our primary study site (implementation). Our implementation team began training the interdisciplinary infectious diseases clinical team in VTH after declaration of the pandemic in March 2020. We pivoted from a randomized clinical trial recruitment and supported modifications in clinic processes by introducing patients to VTH through personalized telephone calls and mailed brochures to inform them of telehealth options during the pandemic. Adaptations were made to provider locations, with some providers delivering care remotely from home and others delivering virtual care from the clinic. We also modified the external and internal facilitator roles to allow external facilitators to provide one-on-one training, troubleshooting assistance, and delivery of necessary equipment.

**Results:** Within 6 weeks of the emergency declaration of the pandemic, 100% of providers (*n* = 27) had conducted at least one appointment, with 24.1% (*n* = 124) of unique patients using VTH. Despite challenges, we capitalized on temporary mandates to assist providers in delivering care virtually. Given our successes, we encourage researchers to be flexible and seek alternative approaches to preserve research efforts in extenuating circumstances. RCT registration: NCT04055207 at clinicaltrials.gov

## Background

The Veterans Health Administration (VHA) plays a critical role in the national HIV/AIDS initiative to end the HIV epidemic, including both innovative research studies and direct clinical care. The focus of our research is improving retention in care for veterans living with HIV.

In early 2020, our team planned to initiate a randomized hybrid type 2 effectiveness–implementation trial^1^ to compare the effectiveness of integrated multidisciplinary HIV care in face-to-face settings to remote care using video telehealth. However, as SARS-CoV-2 spread, resulting in stay-at-home orders, ceasing of nonemergency medical services, and veterans' reluctance to leave their homes, we shifted efforts to support the urgent need to increase capacity for remote clinical care. This allowed us to examine the contextual needs of rapidly implementing video telehealth for HIV care during an emergency.

Despite tremendous advancements in treating HIV, only ∼50% of diagnosed individuals receive recommended routine outpatient HIV care.^2^ Poor retention in routine HIV care is associated with limited access to antiretroviral therapy, leading to lower rates of HIV suppression; higher transmission rates; and, ultimately, poorer survival rates.^3^ Video telehealth to the home (VTH) has equivalent clinical outcomes to in-person care and may address barriers to providing comprehensive care for people with HIV, leading to improved clinical outcomes.^4,5^

As the SARS-CoV-2 pandemic spread and clinical procedures and processes changed, many human subjects research trials were halted, including telehealth studies with an in-person arm such as ours. Our research team pivoted to respond to the immediate needs of the infectious disease (ID) clinic by initiating VTH implementation efforts, rather than waiting to return to normal operations to begin the randomized controlled trial (RCT). Due to existing VHA telehealth infrastructure and our team's ongoing work implementing video telehealth across a variety of clinics,^6^ we felt uniquely positioned to proceed with implementing VTH for HIV care, despite our inability to conduct the planned RCT.

While most providers initially opted for telephone visits as an alternative to in-person care, VTH provides an enriched environment for both provider and patient, allowing both video and audio connectivity, affording a more thorough clinical assessment than a phone call.^7^ VA Video Connect (VVC) is a platform for delivering care to patients across medical and mental health specialties throughout VHA. The need for remote patient management (i.e., virtual health care) has increased the relevance of VVC.

The objective of this article is to describe how our team responded to immediate challenges posed by the first 6 months of the pandemic and highlight lessons learned implementing VTH within a busy ID clinic during a public health crisis while retaining our research focus on data collection and planning to return to the proposed RCT when appropriate.

## Methods

### Overview of original proposed study

Our initial study design involved a randomized trial^8^ (*n* = 360) to compare VTH-delivered multidisciplinary HIV care to standard in-person care to evaluate the impact of virtual visits on retention in care of veterans. For the proposed RCT and consistent with recommended clinical practice, veterans would provide verbal consent to receive VTH-delivered care as an option over in-person care. Our mixed-methods formative evaluation would be ongoing, iterative, and guided by the RE-AIM QuEST framework,^9^ which integrates quantitative and qualitative data, allowing simultaneous data collection of reach among patients, effectiveness of the intervention, adoption among providers, and implementation outcomes (Table 1).

**Table 1.** Proposed RE-AIM QuEST Framework and Modification Outcomes

	Proposed quantitative	Modification outcomes	Proposed qualitative	Modification outcomes
Reach veteran	% of veterans receiving treatment through VTH	24% of veterans (*n* = 124) at the study-site clinic received treatment through VTH	Barriers and facilitators to receiving treatment through VTH (veteran interviews^a^)	Barriers: VVC platform overwhelmed Lack of personal video capable devices and connectivity Facilitators: VHA loaned devices Program Office of Connected Care Help Desk
Effectiveness veteran	Completed at least one visit with a primary care or ID provider in each 6 months of the year at least 60 days apart	NA	Veteran experience with VTH-delivered HIV care (veteran interviews)	NA
Adoption provider	% of providers who agree to provide treatment, complete training, and document treatment through VTH	100% of providers (*n* = 27) conducted at least one visit through VTH 21% (*n* = 131) of all documented encounters were conducted through VTH	Provider experience of delivering HIV care through VTH (provider interviews)	Patient safety Able to contact patients without exposing them Better treatment Allows for a sense of patient's home environment Easier for family members to be involved in care plan Improved retention Address medicine adherence Offload some in-person visits with VTH Importance of visual Video is better for building rapport than telephone Able to see patient attire, ease, facial expressions, and signs of distress Technical issues Frustration with technology glitches Not all patients are trained in VTH
Implementation provider	NA	NA	Contextually specific barriers and facilitators to implementing HIV treatment through VTH	Barriers: Provider telework added complexities to care delivery Minimal clinical space to do VTH Surge in VTH use periodically overwhelmed the platform Facilitators: One-on-one training with research staff Creation of ID clinic SharePoint folder Availability of research staff to troubleshoot for providers
Maintenance provider and system	NA	NA	Opinions of clinicians, stakeholders, and leadership Identify barriers and facilitators affecting sustainability	NA

^a^ Veterans' interviews will be completed at study start. Modification outcomes were assessed by observational feedback from research team outreach.

ID, infectious disease; NA, not applicable; VHA, Veterans Health Administration; VTH, video telehealth to home; VVC, VA Video Connect.

Primary clinical effectiveness outcomes included HIV suppression, adherence to antiretroviral therapy medications, and adherence to counseling, with outcome assessments at baseline and 12 months. Secondary clinical effectiveness data on VTH delivery collected during the RCT could justify further expansion of VTH in HIV care.

### Study modifications

#### System and team readiness to change

As a leader in telehealth, VHA has invested significant resources into telehealth infrastructure, and established successful mental health and primary care telehealth programs. Thus, VHA was poised to transition quickly to telehealth platforms and address changes as needed.

Our team has expertise in personalized implementation of virtual treatments (PIVOT) and formative evaluation.^6,10^ Our approach to implementation is guided by implementation facilitation^11^ and is flexible, allowing us to address the contextual needs of each site that will increase uptake of virtual treatment and improve digital health literacy for both patients and providers.^12^ This flexible implementation strategy has also been deployed in rural settings with success,^10^ demonstrating its utility across multiple contexts.^6^

#### Retained and modified study design

The VHA response to SARS-CoV-2 necessitated multiple modifications to study methodology. The most significant was postponement of the RCT. Randomization in the primary study site was no longer an option, given the shift to virtual care and limited in-person care. The urgent need to limit in-person care and transition to virtual care called for rapidly implementing VTH in the ID clinic at the primary study site, and patients were offered video visits if deemed appropriate by their clinician. For the first 6 months after the declaration of the pandemic in March 2020, our implementation team provided emergency support and began training the interdisciplinary ID clinical team in VTH.

Another adaptation involved external and internal facilitator roles. Typically, external facilitators (research staff) establish relationships with internal facilitators (clinic staff) and both work together in parallel during early stages of implementation. However, with the urgent need to implement virtual care, external facilitators assumed a direct role in implementation, including one-on-one provider training, troubleshooting availability and assistance, delivery of necessary equipment, and advising clinic administrative staff on VTH scheduling and documentation best practices.

Limiting in-person visits required modification of patient interaction. Our original plan included provider introduction of VTH to patients as an option for care, with research staff connecting with patients after in-person clinic visits to support veteran education and use of VTH. Instead, research staff supported the study site's standard practice of telephone and mailed appointment reminders by generating tailored telephone scripts to introduce and assess patient VTH readiness. Research staff also provided clinic staff with printouts of VHA-generated brochures to include with clinic mailers informing patients of VTH options during the pandemic. Brochures, along with other resources (e.g., clinic note templates, documentation guidance, and provider FAQ guides), were kept in a curated ID folder on the pre-existing PIVOT SharePoint site.

We planned to leverage lessons learned from the RCT at the primary study site, and after 12 months expand to six additional sites (other VA medical centers and associated community based outpatient clinics [CBOCs]), focusing on implementation efforts using external facilitators as consultants/trainers for internal facilitators. However, the additional implementation sites were also under pressure to transition from in-person to virtual care. Prioritizing provider and patient care and safety, study PIs advised clinic leadership at the six implementation sites through email not to delay their transition to VTH and connected sites to VHA-generated telehealth resources in support of national mandates to virtualize care.

Guided by RE-AIM Quest frameworks, we sustained the ability to measure aspects of veteran reach and provider adoption, using data from the VHA Support Service Center. Qualitative interviews with study-site providers gave insight into provider adoption and implementation. We were unable to interview veterans as planned, but observations from research staff assessed barriers and facilitators of veteran reach. We were also unable to measure effectiveness and maintenance during this time of implementation support with the study site.

### Challenges of modified implementation

Adapting our research efforts presented significant challenges; however, we were able to gain valuable insight into implementing virtual care in a rapidly changing environment. As nonemergent in-person visits were replaced with virtual visits, the massive influx of users accessing VVC remotely temporarily overwhelmed clinical bandwidth for providers and significantly affected VVC performance. Given these challenges and federal guidance that relaxed HIPAA restrictions during the public health emergency, VHA temporarily approved select commercially available platforms for use to facilitate patient care through VTH and telemedicine.

Before the pandemic, providers expressed ambivalence toward using VTH for connecting with patients for whom the clinic did not provide any of their care with this modality. Given the need to ramp up VTH delivery quickly, we lost our expected ability to build capacity slowly among clinic providers at their individual pace and comfort level, thus likely increasing stress and negative feelings for some clinicians as they were urged to quickly adopt VTH. Our team offered ongoing individualized support to providers through on-demand refresher training, creation of quick start guides, and the ID clinic SharePoint folder.

Adaptations were also made to provider location during the peak of the outbreak, resulting in some providers delivering care remotely from home, while others continued to deliver virtual care from the clinic. Our team trained providers on the differences in delivering care through VTH from their home compared to the clinic, including best practices about their home station setup (e.g., using a tablet as a dual monitor and headphones for sound clarity and privacy) based on VHA telework guidelines.

VHA has a loaned device program that provides veterans with resources to connect with their provider for VTH appointments. As a result of the pandemic, there was a temporary shortage in devices available to veterans. Although our team anticipated VTH serving as a facilitator to reduce barriers to care access, we observed a digital-divide effect that resulted in a potential widening of the gap in care. In response, VHA expanded the existing loaned device program in August of 2020.

Although rapid implementation was more effort intensive for research staff, this model afforded our team more social capital with clinic stakeholders, as well as an opportunity to learn more about nuanced workflow processes within the clinics.

## Results

Table 1 summarizes the proposed outcomes and modification findings. In the emergent 6-month implementation period, 100% of providers (*N* = 27, including 8 MDs, 1 PharmD, 2 physician assistants, and 16 ID fellows) at the study-site ID clinic adopted VTH and delivered care to at least one patient using the modality. During this time, providers at the study site used VTH-delivered care, with 24.1% (124) of unique veterans for 21.9% (131) of all encounters. Although these percentages seem low, the study site ranked third among all VA medical center ID clinics for total VTH encounters.

Qualitative interviews were conducted with study-site providers (*n* = 9, including 7 fellows, 4 MDs, 2 PharmDs, 1 PA, and 1 social worker) regarding barriers and facilitators to provider adoption and implementation. Providers also discussed overall experience with delivering care through VTH. Providers reported on increased patient safety with VTH during the pandemic, better treatment because VTH gives providers a sense of the patients' home environment, improved retention and medicine adherence, the importance of visuals to build rapport, issues with technology not working properly, and lack of patient training. Research staff observation of barriers and facilitators concerning veteran reach took the place of patient interviews.

Table 2 summarizes the demographics (gender, rurality, race, and age) of the veterans at the study site who received care through VTH. The sample reached through VTH is representative of the study-site clinic population. Veterans who used VTH were 94.4% male, 92.7% urban, and 46.8% Black/African American; and 34.7% were 55–64 years old.

**Table 2.** Demographics of Veterans at the Study Site with at Least One Video Telehealth to Home Encounter

Total unique veterans	124, n (%)
Gender	
Female	7 (5.6)
Male	117 (94.4)
Rurality	
Rural	9 (7.3)
Urban	115 (92.7)
Race	
Black/African American	58 (46.8)
White	56 (45.2)
Asian	3 (2.4)
Native Hawaiian/other Pacific Islander	2 (1.6)
Multiple	2 (1.6)
Unknown	2 (1.6)
Declined to answer	1 (0.8)
Age (years)	
<25	2 (1.6)
25–34	9 (7.3)
35–44	20 (16.1)
45–54	18 (14.5)
55–64	43 (34.7)
65–74	21 (16.9)
75–84	10 (8.1)
85+	1 (0.8)

## Discussion

VTH is now a concrete salient concept as a care delivery modality. Meeting providers and patients where they are, addressing their needs, and supporting their clinical efforts, we could explore alternative training approaches (e.g., groups or asynchronous modalities) that might be needed and used in the future. In addition, required modifications to clinical services resulted in more heterogeneous use of VTH and telephone across clinical sites than originally planned.

In response to provider feedback about patients lacking training with the technology, we have determined a need for in-person coaching by research staff to assess veteran technology readiness and boost promotion of provider utilization of the Digital Divide consult in hopes of further reducing digital health disparities. As we plan our return to the proposed RCT in the next 6 months, the addition to the research protocol of an in-person interaction between research staff and veterans requires an IRB amendment as well as approval from the VA associate chief of staff for research of a COVID-19 risk assessment mitigation plan to begin the planned RCT.

## Conclusion

Disruption of clinical research due to extenuating circumstances is not unique to our team or to this pandemic. Therefore, alternative approaches may be considered to preserve research efforts. Implementation scientists could test intentional implementation efforts compared with unsupported mandates during periods of disruption, whereas those focusing on access to care could explore alternative ways to reach out-of-care patients. Teams with a telehealth emphasis might explore leveraging electronic health record applications to facilitate measurement-based care and peripheral devices to promote healthy outcomes.

Overall, the pandemic has increased awareness of the growing need for fundamental change in the way we deliver health care and conduct research, but it highlights the usefulness and adaptability of using technology such as VTH to gather and exchange information. As the proportion of in-person care at the ID clinic has increased, the research team has assessed the feasibility and safety of the RCT and plans to return to the original hybrid type-2 effectiveness–implementation proposed within the next 6 months.

## Abbreviations Used

ID: infectious disease
RCT: randomized controlled trial
VHA: Veterans Health Administration
VVC: VA Video Connect
VTH: video telehealth to home
